# Speckle-type POZ protein functions as a tumor suppressor in non-small cell lung cancer due to DNA methylation

**DOI:** 10.1186/s12935-018-0711-z

**Published:** 2018-12-22

**Authors:** Sumei Yao, Xinming Chen, Jinliang Chen, Yangbo Guan, Yifei Liu, Jianrong Chen, Xuedong Lv

**Affiliations:** 1 Department of Respiratory Medicine, The Second Affiliated Hospital of Nantong University, 6 Haierxiang North Road, Nantong, 226001 Jiangsu People’s Republic of China; 2 Department of Thoracic Surgery, Affiliated Hospital of Nantong University, Nantong, 226001 China; 3 Department of Urology, Affiliated Hospital of Nantong University, Nantong, 226001 China

**Keywords:** DNA methylation, NSCLC, C/EBPα, SPOP, Transcriptional regulation

## Abstract

**Background:**

Tumor suppressor epigenetic silencing plays an important role in non-small cell lung cancer (NSCLC) development and progression. Previously, the expression of speckle-type POZ protein (SPOP) has been found to be significantly inhibited in NSCLC. Our research aimed to investigate the molecular mechanisms, clinical significance and epigenetic alteration of SPOP in NSCLC.

**Materials and methods:**

Bisulfite sequencing PCR and methylation-specific PCR were performed to test gene methylation. Chromatin immunoprecipitation (ChIP) was performed to detect transcription factor C/EBPα combinations and the promoter of the SPOP gene. Furthermore, we evaluated the effects of C/EBPα siRNA on SPOP expression, tumor cell migration and proliferation via MTT and Transwell assays in vitro and tumor growth in vivo. The relationship between the methylation status of the SPOP gene and clinicopathologic characteristics was investigated.

**Results:**

Hypermethylation was found in the CpG island of the SPOP gene promoter in NSCLC tissues, and this methylation was found to be correlated with SPOP expression. SPOP promoter methylation was associated with the pathology grade. The transcriptional activities were significantly inhibited by the hypermethylation of specific CpG sites within the SPOP gene promoter, while 5-aza-2′-deoxycytidine significantly increased SPOP gene expression. C/EBPα also played a key role in SPOP regulation. Five C/EBPα binding sites in the CpG island of the SPOP gene promoter were identified by ChIP. Inhibition of C/EBPα significantly reduced SPOP expression. SPOP mediated the C/EBPα-regulated suppression of invasion, migration and proliferation in vitro and tumor growth in vivo.

**Conclusions:**

SPOP function and expression in NSCLS were regulated by DNA methylation and C/EBPα transcriptional regulation combination effects, indicating that the SPOP promoter methylation status could be utilized as an epigenetic biomarker and that the C/EBPα-SPOP signaling pathway could be a potential therapeutic target in NSCLC.

**Electronic supplementary material:**

The online version of this article (10.1186/s12935-018-0711-z) contains supplementary material, which is available to authorized users.

## Background

Currently, lung cancer is reportedly the leading cause of tumor-related deaths worldwide, accounting for more than 1 million deaths annually regardless of gender or ethnicity [1]. Nearly 85% of all lung carcinomas are non-small cell lung cancer (NSCLC) [2, 3], consisting of lung adenocarcinoma, lung squamous cell carcinoma, lung large-cell carcinoma and other rare types. The NSCLC prognosis is still poor, and the 5-year survival rate is approximately 15% due to several factors, including the small number of effective drugs, low diagnostic rate during the early stage, and high cancer recurrence and metastasis rates [4]. Although NSCLC is among the most extensively studied disorders over the last few years, it is a very complex process that needs further investigation. Similar to other malignancies, the development and progression of NSCLC are multistage processes that involve both epigenetic and genetic changes [5]. As one of the most common epigenetic modifications in mammalian genomes, DNA methylation plays critical roles in tumorigenesis [6]. Alterations in the DNA methylation status subsequently lead to the inactivation of gene expression. Specifically, the inactivation of tumor-suppressor genes caused by hypermethylation of promoter regions has been shown to be important for the onset and progression of cancer [7–10].

Speckle-type POZ protein (SPOP) has been recognized as a novel nuclear speckle-type protein in human cells and an autoantigen in scleroderma patients [11]. Subsequent studies have shown that SPOP acts as an adaptor protein in the CUL3-based E3 ubiquitin ligase complex [12]. Cumulative evidence indicates that ubiquitin–proteasome system dysregulation is involved in the pathogenesis of cancer. Ubiquitin binds its target proteins through a cascade enzyme system that consists of E1 activating enzyme, E2 conjugation enzyme and E3 ubiquitin ligase, and their substrate specificity is conferred by E3 ubiquitin ligase [13, 14]. Among the E3 enzymes, Cul3-based ligase has recently been demonstrated to participate in many cellular processes, such as protein trafficking, cell cycle regulation, stress responses and development [15]. Recent studies have firmly established that SPOP is a substrate-specific adaptor that binds Cul3 and exerts cancer-promoting or cancer-suppressive effects depending on the specific substrate in various cancers [16, 17]. Somatic mutation analyses of cancer genomes have indicated that the SPOP gene is always mutated in several human cancers; for example, approximately 10% of prostate cancers [18], 8% of endometrial carcinoma [19] and 2.2% of colorectal cancer [20] reveal SPOP gene mutations. However, the regulation mechanism of SPOP expression in NSCLC is still unclear [21]. Intriguingly, our pre-experiment found that SPOP was obviously inhibited in NSCLC tissues compared with that in adjacent normal lung tissues [22], suggesting that SPOP functions as a cancer suppressor gene (TSG). Additionally, Kim et al. revealed that SPOP somatic mutations were not harbored in solid tumors, indicating that somatic mutations and decreased SPOP expression might be separate events in NSCLC [20]. As mentioned above, aberrant TSG DNA hypermethylation is a hallmark of many human cancers [23]. Taken together, we speculated that SPOP gene hypermethylation could be associated with lung cancer pathogenesis.

C/EBP alpha (C/EBPα) is one of the basic member of C/EBP family and was first identified in nuclear extracts of rat liver for its ability to bind the CCAAT box. As a well-known transcription factor, C/EBPα induces the transcription of several lineage-specific genes, such as G-CSF-R [24] and PPARγ [25]. Multiple studies demonstrated that C/EBPα involved in human cell proliferation and migration [26, 27]. However, the mechanism how C/EBPα inhibits tumor progression is still unclear.

Our study aimed to investigate the SPOP gene methylation status in NSCLC and determine the relationship between the SPOP gene methylation level and clinicopathological parameters of NSCLC patients. Furthermore, we demonstrated that SPOP is transcriptionally activated by C/EBPα, while CpG island methylation in the promoter region of SPOP leads to C/EBPα binding abrogation. Additionally, SPOP was found to mediate the C/EBPα-regulated suppression of NSCLC cell proliferation, invasion and migration in vitro and tumor growth in vivo.

## Materials and methods

### Tissue samples and clinical data

Tumor specimens and matched adjacent non-tumor tissues (at least 5 cm away from primary tumor tissue) were collected by a pathologist from 82 NSCLC patients undergoing surgical resection at the Second Affiliated Hospital of Nantong University and the Affiliated Hospital of Nantong University between January 2004 and January 2010. After the surgical removal, the tissue samples were immediately frozen in liquid nitrogen and maintained at − 80 °C until they were used for the real-time RT-PCR, methylation and western blot analyses; meanwhile, parts of the specimens were formalin-fixed and paraffin-embedded for the immunohistochemical staining and histopathologic diagnosis. The clinical features of the patients, including their age, gender, smoking history, and so on, were also collected (Table 1). The study protocol was approved by the Ethics Committee of the Second Affiliated Hospital of Nantong University and the Affiliated Hospital of Nantong University, and all experiments were performed in accordance with the approved guidelines of the Second Affiliated Hospital of Nantong University and the Affiliated Hospital of Nantong University. Written informed consent was obtained from the patients for the publication of this study and any accompanying images. All NSCLC patients underwent standard surgery for maximal tumor resection. No patients received radiotherapy, chemotherapy, molecule-targeted treatment or immunotherapy prior to surgery. The clinical stages and tumor histological grades were evaluated based on the pathological results after surgery. The histological diagnoses of the tumors were evaluated according to the 4th Edition of the World Health Organization Classification. All tumors were staged based on the pathological tumor/node/metastasis (pTNM) classification (7th edition) of the International Union Against Cancer.

**Table 1:** Clinicopathological characteristics and SPOP methylation in 82 NSCLC patients

Clinicopathological characteristics	Number (n = 82)	SPOP methylation			P
		Methylated (n = 33)	Partial methylation (n = 28)	Unmethylated (n = 21)	
Age (years)					0.448
≤ 60	44	15	16	13	
> 60	38	18	12	8	
Gender					0.416
Male	48	22	14	12	
Female	34	11	14	9	
Histologic type					0.086
Adenocarcinoma	63	29	21	13	
Squamous cell carcinoma	19	4	7	8	
Smoking status					0.064
Ever	34	9	16	9	
Never	48	24	12	12	
Staging					0.903
I + II	60	25	20	15	
III + IV	22	8	8	6	
Pathology grade					0.018*
Well	14	3	2	9	
Moderate	43	20	15	8	
Poor	25	10	11	4	
Lymph node metastasis					0.094
No	45	23	13	9	
Yes	37	10	15	12	

* Indicates statistically significant difference

### Cell lines and cell culture

The lung adenocarcinoma cell lines A549, H1299, and A427 and the normal bronchial cell line 16HBE were used. All cell lines were cultivated in standard R10 media (RPMI1640, Invitrogen, Karlsruhe, Germany) at 37 °C in a humidified atmosphere of 5% CO_2_ in air. The medium was supplemented with 10% fetal bovine serum (FBS) (v/v), 50 U/mL penicillin, and 50 μg/mL streptomycin; all obtained from Invitrogen (Karlsruhe, Germany).

### Methylation-specific PCR

The CpG island methylation status of the NSCLC tissue and cells was initially screened at the SPOP gene promoter regions by methylation-specific PCR (MSP). The extracted sample DNA was modified with bisulfite reagents according to the instructions of the manufacturer (Zymo Research, CA). This modification converted unmethylated cytosine to thymine, while methylated cytosine remained unchanged. In total, 20 ng of bisulfite-modified DNA were subjected to PCR amplification and directly sequenced using an ABI3700 automated sequencing system (Applied Biosystems, CA). If the CpG sites in the region analyzed by MSP are methylated, a methylated (M) band appears. However, the unmethylated (U) band appears when the sites are unmethylated. Occasionally, both bands could be present if the sites are partially methylated. The following MSP primers were designed for SPOP: methylated: SPOP-MSP-M-F: 5′-TTTCGGAGTAGTTGGGATTATAGAC-3′, SPOP-MSP-M-R: 5′-CGAAAACTAAAACAAAAAAATAACGA-3′, and unmethylated: SPOP-MSP-U-F: 5′-TTTTTGGAGTAGTTGGGATTATAGAT-3′, SPOP-MSP-U-R: 5′-AAAAACTAAAACAAAAAAATAACAAA-3′.

### Bisulfite sequencing PCR (BSP)

The status of methylation was further investigated by bisulfite sequencing PCR (BSP). Bisulfite-treated DNA was amplified by PCR using the following primers: SPOP-BSP-F: 5′-TGTTGTGAAATGTTTTTAATTGTTT-3′, SPOP-BSP-R: 5′-CCTAAATTTCCCTACTCCTCCTC-3′. The PCR products were purified with a TIANgel Midi Purification Kit (TiangenBiltech Co. Ltd). Then, the PCR products were cloned into a pGEM-T easy vector (Promega, Madison, WI, USA). Ten colonies were randomly chosen for the plasmid extraction of DNA using a PromegaSpin Mini kit (Promega) and then sequenced by an ABI 3130 Genetic Analyzer (Applied Biosystems, Foster City, CA, USA).

### Western blotting

A bicinchoninic acid (BCA) kit (Sigma-Aldrich, St. Louis, USA) was used to test the protein concentration. The proteins were boiled at 95 °C for 10 min after adding sample buffer to the proteins (each well, 30 µg per sample). Then, the proteins were separated using 10% polyacrylamide gel electrophoresis. The proteins were transferred to polyvinylidene fluoride membranes with 100 V transfer-molded voltage for 45 to 70 min after electrophoresis. Then, the samples were incubated at room temperature for 1 h with primary antibodies (1:1000 dilution) and 5% bovine serum albumin (BSA) at 4 °C overnight. The samples were washed with tris-buffered saline Tween 20 (TBST) 3 times (5 min/time). The corresponding secondary antibody was added, and the samples were incubated at room temperature for 1 h. Then, the membranes were washed 3 times (5 min/time). The proteins were detected using chemiluminescence reagents. GAPDH was used as an internal reference. The bands were visualized with Bio-Rad Gel Doc EZ imager (Life Science Research, California, USA). Image J software (National Institutes of Health, Maryland) was applied to analyze the intensity of the target bands.

### Quantitative real-time PCR

Quantitative real-time PCR (qRT-PCR) was used to detect the SPOP expression. The total RNA was extracted with TRIzol reagent (Invitrogen, Life Technologies Corporation, 3175 Staley Road, Grand Island, NY 14072 USA) following the instructions of the manufacturer. In total, 1 µg RNA was subjected to reverse transcription using a PrimeScript RT reagent kit (Takara, Japan). The conditions of the reverse transcription were as follows: 1 cycle at 37 °C for 15 min and 1 cycle at 85 °C for 5 s. Then, the diluted cDNA (2 µL) was used for the real-time PCR analysis using SYBR Green I (Takara, Japan) along with primers synthesized by Nanjing GenScript Co., Ltd. (Nanjing, Jiangsu, China). The primer sequences were as follows: SPOP forward 5′-AAGGGTTAGATGAAGAAAGCA-3′ and SPOP reverse 5′-TGCCCGAACTTCACTCTT-3′. The following conditions were used for the real-time fluorescent quantitative PCR analysis: 1 cycle at 95 °C for 5 min during the holding stage; 40 cycles at 95 °C for 15 s and 60 °C for 60 s during the cycling stage; and 1 cycle at 95 °C for 15 s, 60 °C for 1 min and 95 °C for 15 s during the melt curve stage. The thermal cycling and real-time detection were conducted using StepOnePlus Real-Time PCR Systems (ABI, Life Technologies Corporation, 3175 Staley Road, Grand Island, NY 14072 USA). The quantities of each mRNA were calculated using the comparative (2^−ΔΔCt^) method.

### MTT assay

During the logarithmic growth phase, the cells were washed twice with PBS, detached by trypsin and made into single cell suspensions. A cell analyzer was used for the cell counting. The cells were inoculated into a 96-well plate at 3 × 10^3^–6 × 10^3^ cells/well (200 μL per well, 6 duplicated wells) and incubated for 24–72 h in a 5% CO_2_ incubator at 37 °C. Then, 20 μL MTT solution were added to each well. The plate was incubated in a 5% CO_2_ incubator at 37 °C for another 4 h. Subsequently, the incubation was ended, and the solution was removed. DMSO (150 μL) was added to each well, and the samples were shaken for 10 min to dissolve the crystal. An enzyme-linked immunosorbent assay instrument was used to detect the optical density (OD) value of each well at 12 h, 24 h, 48 h and 72 h. An MTT curve graph was constructed with the OD value set as the ordinate and the interval time set as the abscissa. The experiment was repeated 3 times.

### Drug treatments

To determine the methylation regulation of SPOP gene expression, the A427 and H1299 cell lines were treated with the DNA-demethylating agent 5-aza-2′-deoxycytidine (AZA; Sigma-Aldrich). For dose–response experiments, the cultured cells were treated with AZA at 0.5, 1.0, 1.5, 2.0, and 2.5 μM for 4 days. The drug containing medium was changed every day. After the drug treatments, the cells were washed with PBS and harvested to measure gene and protein expression.

### Plasmid construction, siRNA and transfection

Human genomic DNA was isolated from 16HBE cells using a genomic DNA isolation kit (TiangenBiltech Co. Ltd) according to the instructions of the manufacturer. In total, a 1.3 kb fragment of the 5′-flanking sequence of SPOP was amplified using ProbestTaq DNA polymerase (Takara, Japan). The NheI and XhoI restriction sites were integrated into the fragment. The fragment was cloned into plasmid pGL3-Basic vectors named pGL-C5 (− 1208/+ 110), pGL-C4 (− 932/+ 73), pGL-C3 (− 720/+ 73), and pGL-C2 (− 312/+ 73), which were created by PCR. Three site-directed mutant constructs were generated at potential C/EBPα binding sites (pGL3-MT1, pGL3-MT2, and pGL3-MT5) based on the pGL3-C5 structure using the PCR-based Megaprimer technique as previously described [28]. All plasmids were verified by sequencing (ABI PRISM^®^ 3100 Genetic Analyzer; Applied Biosystems).

The SPOP (NM_001007226.1) full-length ORF was amplified from cDNA from 16HBE cells and cloned into a pCDNA3.1 vector, Similarly, the C/EBPα (1035 bp, NM_001287435.1) full-length ORF was amplified from cDNA from 16HBE cells and cloned into a pCDNA3.1 vector. siRNAs targeting human SPOP (siSPOP), C/EBPα (siC/EBPα) and a non-targeting control siRNA (siControl) were purchased from RiboBio Co., Ltd. (Guangzhou, China). For the gain or loss of gene expression analyses in lung cancer cells, the cells were transiently transfected with the gene expression plasmid and vector control using Lipofectamine 2000 (Invitrogen, Carlsbad, CA) according to the instructions of the manufacturer. The siRNA transfection was performed using (Invitrogen) 20 nM based on the protocol of the manufacturer.

### Dual-luciferase reporter assay

The cells were plated at a density of 2 × 10^5^ cells/well in 24-well plates for an analysis of promoter activation. siC/EBPα or siControl was cotransfected with the SPOP promoter constructs and the internal control pRL-TK. The firefly and Renilla luciferase activities were measured 48 h after transfection using a Dual-Luciferase reporter assay system (Promega). The relative promoter activation is represented as a ratio of firefly to Renilla luciferase activity.

The CpG island region of SPOP (− 272 to − 27) and SPOP (− 160 to − 27) were amplified from the genomic DNA of 16HBE cells to investigate the influence of CpG island methylation on SPOP gene promoter activity. The amplified promoter fragments were gel-purified as described above and cloned into pGL3-Basic vectors named pGL3-272 and pGL3-160. The enriched fragments were removed from pGL3-Basic and methylated in vitro using SssI methylases (New England Biolabs) or no enzymes (mock) based on the protocol of the manufacturer. The methylation efficiency was confirmed by restriction enzyme digestion using HhaI and HpaII (New England Biolabs). The methylated or mock-methylated fragments were religated into pGL3-Basic to assess the differentially methylated promoter activity.

### Chromatin immunoprecipitation assay

The chromatin immunoprecipitation (ChIP) assay was performed using a Chromatin Immunoprecipitation Kit (Sigma) according to the protocol. Briefly, the tissues were first cut into small pieces, and the protein-DNA complexes were cross-linked with 1% formaldehyde, followed by nuclear fractionation and DNA shearing through sonication. The immunoprecipitation was performed using an anti-CEBPα antibody (ab40764, Abcam) or mouse immunoglobulin G (IgG, negative control). The antibody-protein-DNA complex was eluted from the beads and reversed cross-linked after washing. The purified DNA was subjected to PCR using primers specific to the human SPOP gene promoter after removing the protein and RNA. The following PCR primers were designed and synthesized by GeneChem (Shanghai, China): P1-F: 5′-TGTTAGCCAGGATGGTCTCG-3′ and P1-R: 5′-GCCTGGATGGCAGAGCA-3′ (163 bp); P2-F: 5′-GCGATCTCGGCTCACTG-3′ and P2-R: 5′-AATTAGCTGGGCGTGGC-3′ (240 bp); P3-F: 5′-TAGGGTTTCGCCATGTTGG-3′ and P3-R: 5′-CCACGCATGGTGGCTGA-3′ (110 bp); P4-F: 5′-AGACCTAAATCGCTGTTGTGA-3′ and P4-R: 5′-TGGGTTTCCCTGCTCCT-3′ (256 bp); and P5-F: 5′-AGGAGCAGGGAAACCCA-3′ and P5-R: 5′-TCATACTGTCCGCAACATCC-3′ (229 bp). The PCR conditions were as follows: 95 °C for 5 min, (95 °C for 30 s, 55–60 °C for 30 s, and 72 °C for 30 s) × 36 cycles, and 72 °C for 10 min. The products were visualized using agarose gel electrophoresis.

### Migration and invasion assay

Subsequently, 5 × 10^4^ cells were plated in 8.0-μm pores in a 24-well plate chamber insert (Corning, Corning, NY) with serum-free medium on the top of the insert and medium containing 10% FBS at the bottom of the insert for the cell migration assay. The cells were incubated for 24 h and fixed with 4% paraformaldehyde for 15 min. The cells on the top of the insert were scraped with a cotton swab after washing with PBS. The cells adherent to the bottom were stained with 0.5% crystal violet blue for 15 min at room temperature and then washed with ddH_2_O. The positively stained cells were examined under a microscope. Corning BioCoat GFR Matrigel Invasion Chambers were used instead of chamber inserts in the migration assay to determine cell invasion. The steps described above were performed for the cell invasion assay.

### Tumor xenografts

This experimental protocol was approved by the Research Ethics Committee of Nantong University. Male BALB/c nude mice (5 weeks old) were housed under a 12-h light–dark cycle in a temperature-controlled room (25 ± 1 °C). The mice had ad libitum access to tap water and standard laboratory chow. In total, 24 mice were randomly divided into four groups. siControl + vector, siC/EBPα + vector, siControl + SPOP, or siC/EBPα + SPOP transfected cells were injected subcutaneously into the flanks of the mice (10^6^ cells/100 μL per flank). The tumor diameters were measured every other day with a vernier caliper in two dimensions and calculated as the tumor volume V = d^2^ × D/2 (where d is the tumor measurement at the shortest point, and D is the tumor dimension at the longest point). The mice were sacrificed, and the tumors were removed and photographed after 4 weeks.

### Statistical analysis

All data were analyzed by GraphPad Prism version 6 statistical software. The measurement data are expressed as the mean ± standard deviation. A t-test was used for comparisons between two groups. One-way analysis of variance was applied for comparisons among multiple groups. Statistical significance was assumed at *P *< 0.05.

## Results

### SPOP expression levels in NSCLC tissues are inversely correlated with the CpG island methylation extent in the promoter region

**Fig. 1:**
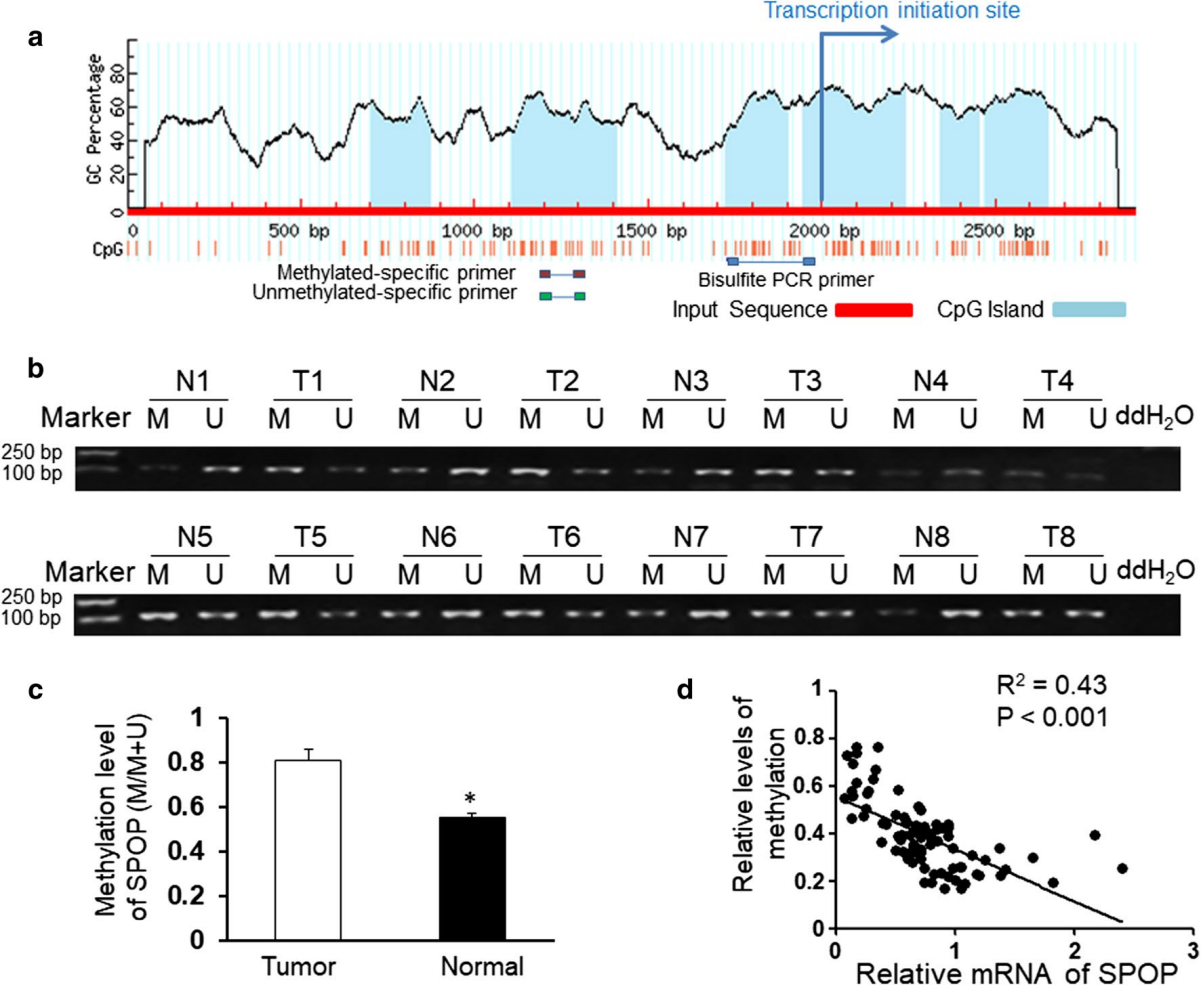
Hypermethylation of the CpG island in the SPOP gene promoter in NSCLC. **a** Scheme of the location of the CpG islands in the promoter of the SPOP gene. The CpG sites are indicated by vertical red lines. The regions used for MS-PCR and BSP are also indicated. **b** Representative results of methylation analysis by MS-PCR in NSCLC tissues (T) and adjacent normal lung tissues (N). U unmethylation; M, methylation. **c** Statistical results showing that the methylation level in the NSCLC tissues was higher than that in the adjacent normal tissues. **d** Correlation analysis of the MSP results. *P < 0.05. Data represent the results of three independent experiments

Our previous study found that the expression of SPOP was significantly inhibited in NSCLC tissues compared with that in normal tissues [22]. To determine whether the low SPOP expression in NSCLC is caused by DNA hypermethylation, our study searched the Human Genome Database for the presence of CpG islands in the SPOP promoter using Online software (http://www.urogene.org/methprimer) and found some CpG islands in the vicinity of the transcription start site (Fig. 1a). We further ana-lyzed the CpG island methylation status in the SPOP promoter in NSCLC tissues and adjacent normal lung tissues from 82 patients with NSCLC by using MSP. The methylation level in the promoter of the SPOP gene in the NSCLC tissues was higher than that in the adjacent normal lung tissues (*P *< 0.05, Fig. 1c), which is consistent with the low SPOP expression in NSCLC tissues. Representative MSP results of eight cases are presented in Fig. 1b. The QPCR indicated that a negative correlation exists between the SPOP promoter methylation degree and SPOP gene expression in NSCLC tissues (Fig. 1d). The MSP of NSCLC showed that SPOP promoter methylation was found in 61 of the 82 NSCLC patients and was associated with the pathology grade, while no correlation was found between the SPOP promoter methylation extent and age, gender, histologic type, smoking status, staging, and lymph node metastasis (Table 1).

### Specific CpG site hypermethylation within the SPOP promoter inhibits transcriptional activities

**Fig. 2:**
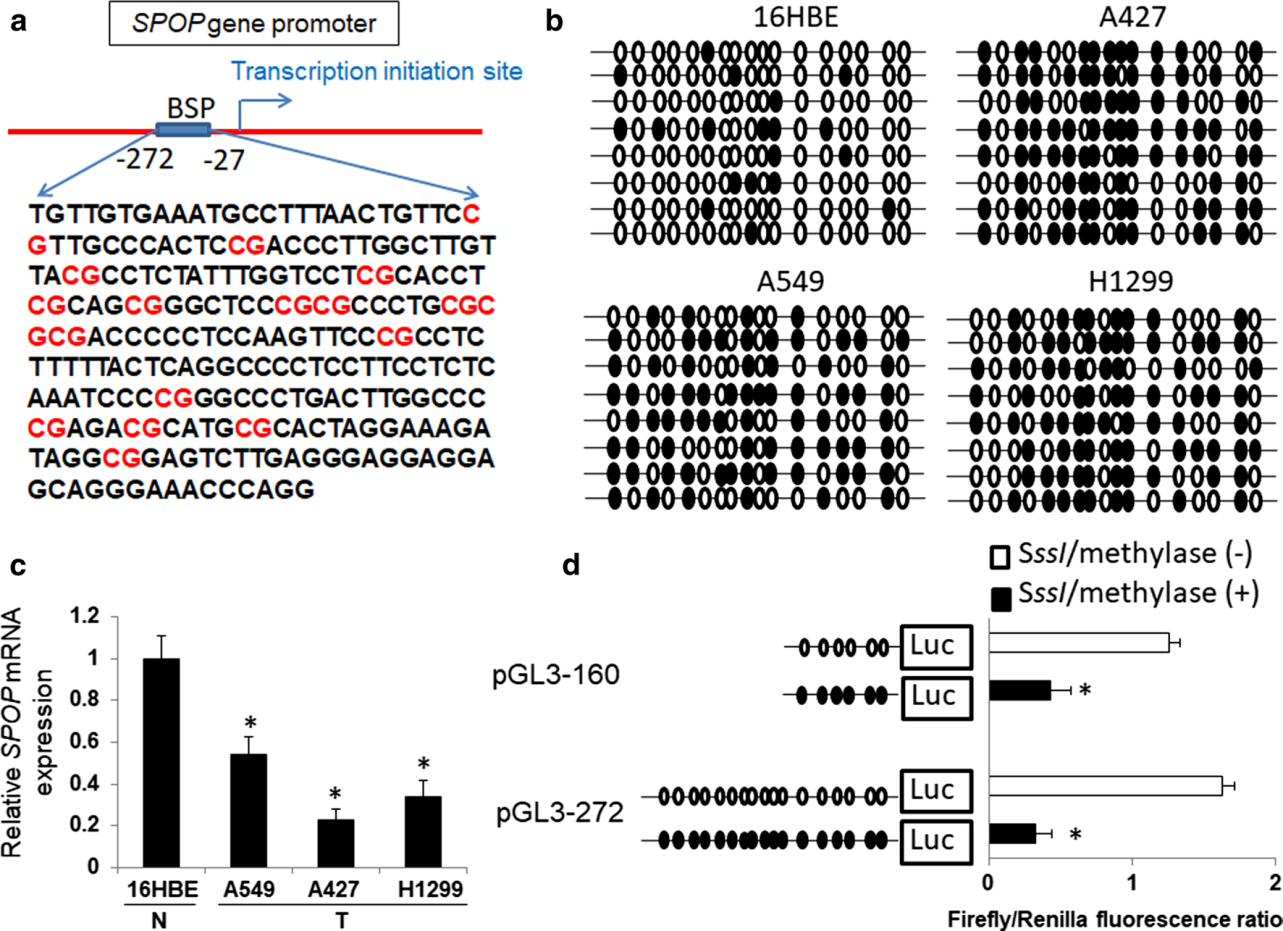
Hypermethylation of specific CpG sites within the SPOP gene promoter inhibits transcriptional activities. **a** Schematic of the regions used for BSP showing the locations of the 17 CpG sites in the SPOP gene promoter area. **b** Bisulfite sequencing analysis of the methylation status of the SPOP gene promoter. Each oval indicates clones from different cell lines. Eight clones were subjected to bisulfite sequencing. The methylated clones of individual CpG sites are labeled in black. **c** The mRNA levels of SPOP in different cell lines. **d** Treatment with two luciferase constructs with SssI methylase in vitro. *P < 0.05. Data represent the results of three independent experiments

To determine whether CpG island methylation is involved in silencing SPOP expression, the DNA methylation status and SPOP expression levels in the lung cancer cell lines A549, A427, and H1299 and the normal human bronchial cell line 16HBE were examined by BSP and qPCR. The BSP detection region contains 17 CpG sites in the CpG island of the SPOP gene promoter region (Fig. 2a). The results indicated that the SPOP promoter region was highly methylated in the lung cancer cell lines A549, A427 and H1229 but lowly methylated in the normal bronchial cell line 16HBE (Fig. 2b). The SPOP mRNAs were consistently expressed at low levels in the A549, A427 and H1299 cells but at high levels in the 16HBE cells (Fig. 2c).

Two SPOP gene promoter region constructs containing the CpG island were treated with *SssI* methylase in vitro and transfected into 16HBE cells (Fig. 2d) to investigate which CpG site is responsible for SPOP methylation-associated inactivation. Compared with the untreated constructs, the increased promoter construct methylation induced significant promoter activity repression. Compared with PGL3-160, the PGL3-272 region failed to affect the promoter activities with or without the SssI methylase treatment. The data revealed that an increase in the DNA methylation level at the region (− 160 to − 27) of the SPOP promoter is an important factor for SPOP transcription regulation.

### SPOP expression is induced by AZA treatment in methylated lung cancer cells

**Fig. 3:**
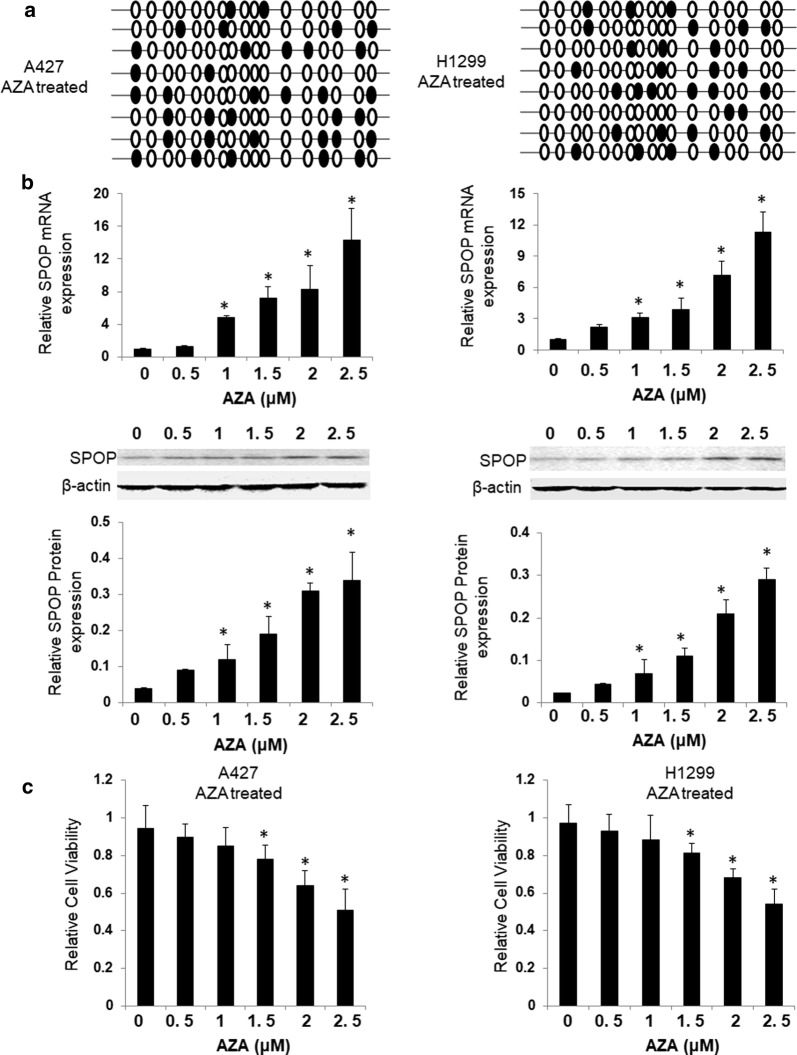
SPOP expression is induced by AZA treatment in methylated lung cancer cells. **a** DNA methylation status of the SPOP gene proximal promoter was analyzed by bisulfite sequencing in AZA-treated cells. **b** SPOP mRNA and protein levels were determined by qPCR and western blotting in the indicated cells treated with varying concentrations of AZA. **c** Effect of AZA on lung cancer cell growth. MTT assay was used to detect the growth of cell treated with different concentrations of AZA. The data are presented as the mean ± SD of three independent experiments. *P < 0.05 as compared with control (Non-treated with AZA)

The lung cancer cell lines were treated with the DNA methyltransferase (DNMT) inhibitor 5-Aza-2′-deoxycytidine (AZA) at concentrations of 0, 0.5, 1.0, 1.5, 2.0, and 2.5 μM, and the SPOP expression levels were subsequently measured by qPCR and western blotting to further determine the DNA methylation effect on SPOP expression. The gene methylation degree in the cell lines A427 and H1299 was higher than that in the cell line A549; thus, the A427 and H1299 cells were selected as research objects. The AZA demethylation effect on the SPOP CpG island in the highly methylated A427 and H1299 cells was confirmed by bisulfite sequencing (Fig. 3a). AZA elicited a dose-dependent induction of SPOP mRNA and protein expression in the highly methylated A427 and H1299 cells (Fig. 3b). These results indicate that SPOP promoter region methylation leads to SPOP suppression in lung cancer cell lines. MTT assay was used to detect the growth of cell treated with different concentrations of AZA. We found that AZA significantly inhibited the growth of A429 and H1299 cells in a dose-dependent manner (Fig. 3c).

### C/EBPα binding to the SPOP gene promoter region is abrogated by DNA methylation

**Fig. 4:**
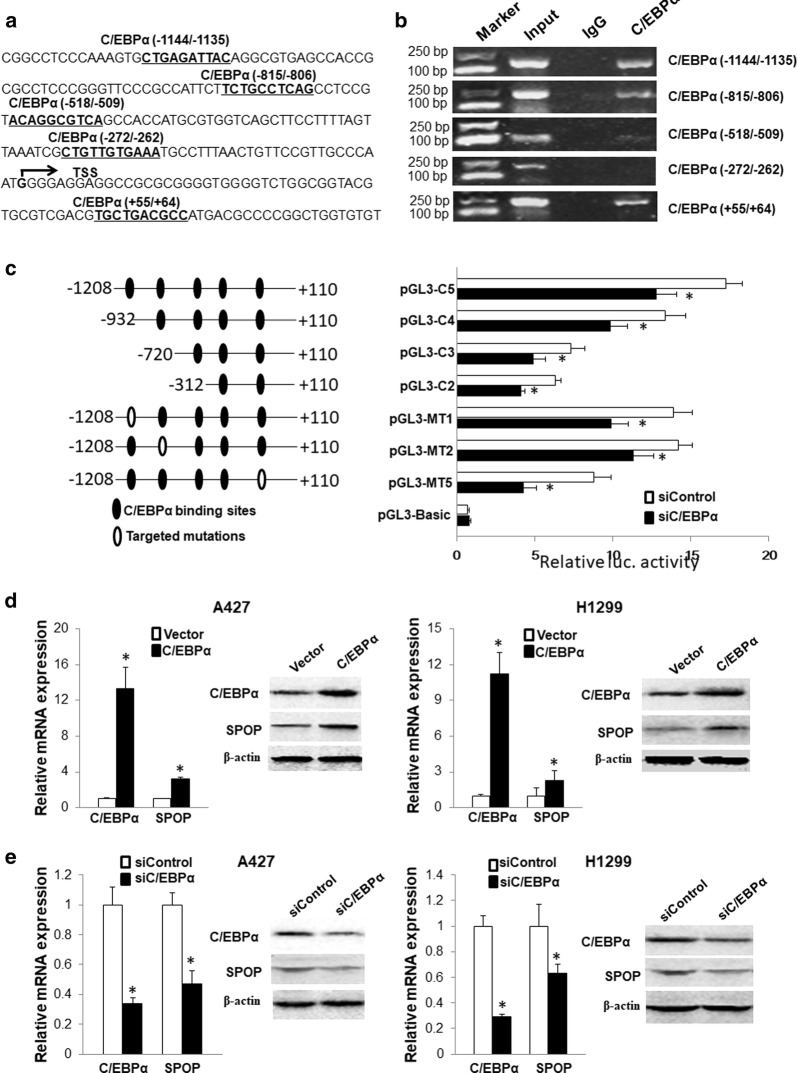
C/EBPα binding the SPOP proximal promoter region is abrogated by DNA methylation and induces SPOP expression in lung cancer cells. **a** Location of the predicted C/EBPα binding sites in the SPOP gene promoter region. The sequences depicted in boldface and underlined denote the predicted binding sites of C/EBPα, and the TSS is labeled. **b** ChIP assay demonstrating the direct binding of C/EBPα to the SPOP gene promoter in NSCLC tissues. The ChIP-enriched DNA fragments of the SPOP gene promoter using IgG and an anti-C/EBPα antibody were amplified by PCR. Total input was used as a positive control. **c** Sequential deletion and substitution mutation analyses identified C/EBPα binding sites in the SPOP gene promoter region. Serially truncated and mutated SPOP gene promoter constructs were cotransfected with siC/EBPα or siControl into A427 cells, and the relative luciferase (*luc.*) activities were determined. Following the transfection of the C/EBPα expression plasmid (**d**) or siC/EBPα (**e**) into the A427 and H1299 cells, the C/EBPα and SPOP mRNA and protein levels were determined by qPCR and western blotting. The data are presented as the mean ± SD of three independent experiments. *P < 0.05

To determine whether C/EBPα is involved in SPOP expression regulation, we first analyzed the correlation between C/EBPα and SPOP expression by bioinformatics. Based on the TCGA data, the expression of SPOP (Additional file 1: Figure S1A) and C/EBPα (Additional file 1: Figure S1B) in lung adenocarcinoma and lung squamous cell carcinoma has a downward trend compared with that in normal tissues. The correlation analysis indicated that the C/EBPα and SPOP alterations were significantly positively correlated in lung adenocarcinoma (P < 8.9e−14, Additional file 1: Figure S1C) and squamous cell carcinoma (*P *< 2.3e−05, Additional file 1: Figure S1D). Furthermore, we performed an online search for the prediction of transcription factor binding sites in the SPOP gene promoter region using online software (http://gene-regulation.com/pub/programs/alibaba2/index.html) and found five putative binding sites for transcription factor C/EBPα located at − 1144 to − 1135, − 815 to − 806, − 518 to − 509, − 272 to − 262, and + 55 to + 64 (Fig. 4a). A ChIP assay was performed using an anti-C/EBPα antibody in the NSCLC tissues. The anti-C/EBPα antibody-enriched DNA sequences of the five regions containing the putative C/EBPα-binding sites were amplified by PCR to confirm the binding of C/EBPα to the predicted sites in vitro. The results indicated that C/EBPα clearly bound the SPOP gene promoter at − 1144 to − 1135, − 815 to − 806 and + 55 to + 64, while no binding was observed at the other predicted sites (Fig. 4b).

Serially truncated and mutated SPOP gene promoter plasmids were constructed, and a luciferase reporter assay was performed in the presence of siControl or siC/EBPα to determine which binding site is functionally required for C/EBPα-regulated SPOP gene promoter activation. pGL3-C5, which contains the five putative C/EBPα binding sites, showed maximum promoter activity, while the cells transfected with siC/EBPα exhibited a prominent reduction in promoter activity. The deletion of the region containing only the − 312 to + 110 site (pGL3-C2) also caused a significant decrease in SPOP gene promoter activity with C/EBPα inhibition. The deletion constructs pGL3-C3 and pGL3-C4 in the siC/EBPα-transfected cells also revealed lower promoter activity at levels similar to those observed in the control cells, which expressed endogenous C/EBPα (Fig. 4c). To determine whether the three identified C/EBPα binding sites play a role in the transcriptional activation of SPOP, we individually generated substitution mutations of the sites (pGL3-MT1, pGL3-MT2, and pGL3-MT5). A significant reduction in SPOP gene promoter activity was found when the − 1144 to − 1135, − 815 to − 806 and + 55 to + 64 sites were individually mutated. Consistent with the data obtained using the deletion mutants, the abrogation of the individual sites resulted in a small decrease in SPOP promoter activation (Fig. 4c).

### C/EBPα is involved in SPOP expression regulation in lung Cancer

The C/EBPα expression plasmid or C/EBPα siRNA was transiently transfected into A427 or H1299 cells, respectively, to investigate the role of C/EBPα in SPOP expression regulation in lung cancer cells. The C/EBPα overexpression significantly increased the SPOP mRNA and protein expression (Fig. 4d), while the C/EBPα inhibition significantly reduced SPOP expression in the A427 and H1299 cells (Fig. 4e). Taken together, these results reveal that C/EBPα is involved in SPOP expression transcriptional regulation.

### SPOP mediates C/EBPα-regulated proliferation, migration and invasion of lung cancer cells

**Fig. 5:**
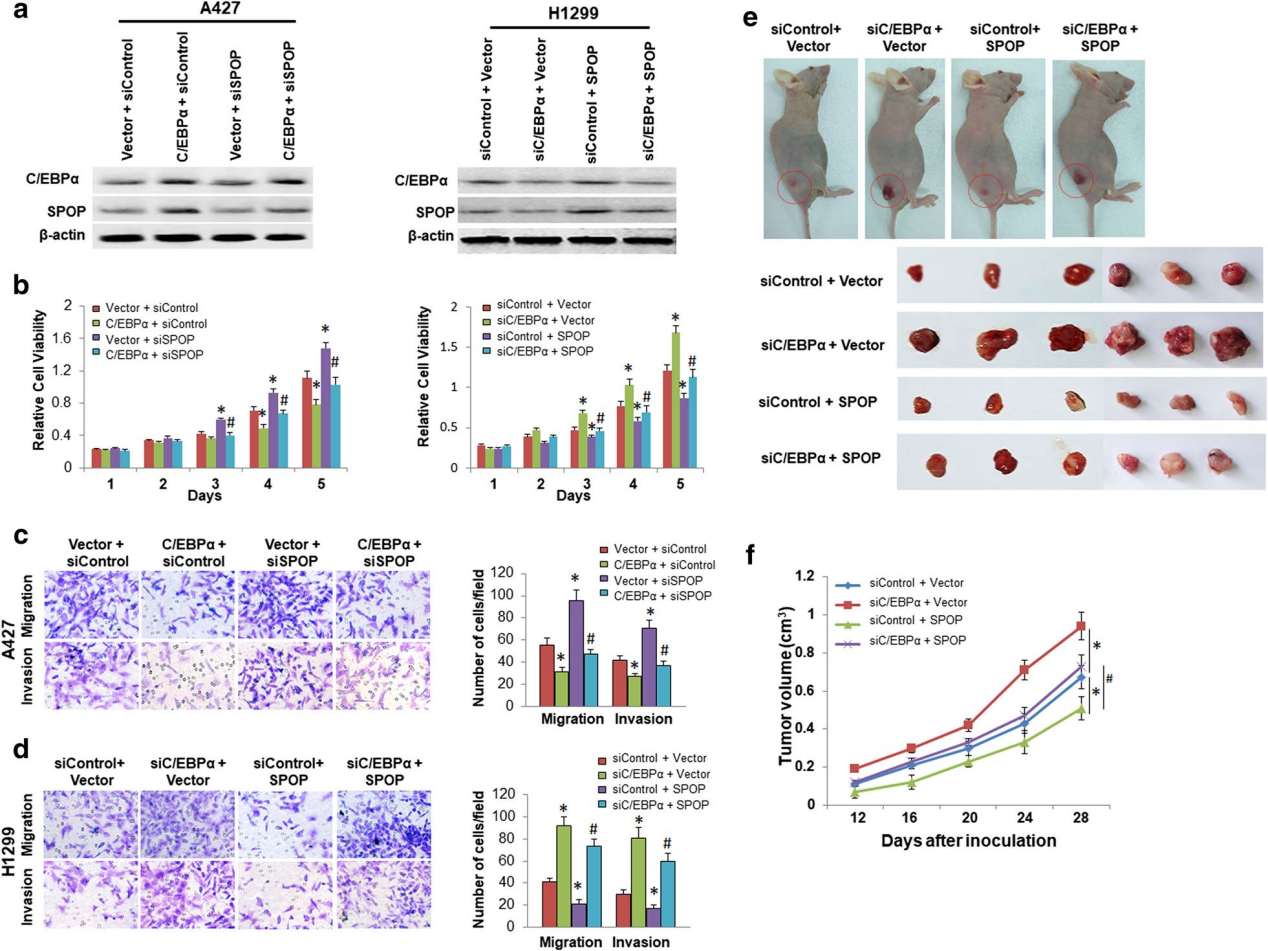
SPOP mediates C/EBPα-regulated proliferation, migration and invasion of lung cancer cells in vitro and in vivo. **a** C/EBPα and SPOP expression in A429 and H1299 cells treated as indicated was detected by western blotting. **b** Cell proliferation ability was assessed by MTT assays in A429 and H1299 cells treated as indicated. **c**, **d** Cell Migration and invasion abilities were assessed using Transwell and Matrigel-coated Transwell assays, respectively, in A427 (**c**) and H1299 (**d**) cells treated as indicated. The data are presented as the mean ± SD of three independent experiments. *P < 0.05 versus A427 or H1299 cells cotransfected with siRNA and vector control (siControl + Vector); ^#^P < 0.05 versus A427 cells cotransfected with siSPOP and vector control (Vector + siSPOP) or H1299 cells cotransfected with siControl and SPOP expression plasmid (siControl + SPOP). **e** Representative xenograft tumors in nude mice (upper left panel), and representative photographs of tumors removed from bodies 28 days after implantation (bottom left panel). **f** Tumor growth curves showing that the C/EBPα depletion significantly enhanced xenograft tumor growth, and these effects were reversed by the SPOP overexpression in the nude mice. *P < 0.05 versus siRNA and vector control group; ^#^P < 0.05 versus siSPOP and vector control group

C/EBPα has been implicated as a tumor suppressor in lung cancer [29]. Previous studies have reported that SPOP plays a role in cell growth suppression in NSCLC cells; therefore, we speculated that SPOP mediates the C/EBPα-suppressed proliferation in lung cancer cells. Thus, the C/EBPα expression plasmid was cotransfected with siSPOP or siControl into A427 cells, and the cell proliferation ability was assessed using an MTT assay. The results indicated that the C/EBPα overexpression increased SPOP expression and inhibited cell proliferation, and these effects were reversed by the siSPOP transfection (Fig. 5a). Conversely, when the H1299 cells were cotransfected with siC/EBPα and the SPOP expression plasmid or vector control, the increased cell proliferation by the C/EBPα depletion was reversed by the SPOP expression plasmid transfection (Fig. 5b). The C/EBPα depletion also inhibited SPOP expression in the H1299 cells (Fig. 5b). On the contrary, we removed C/EBPa and added back SPOP in H1299 cells, and removed SPOP and added back C/EBPa in A427 cells. We found similar effects on cell growth (Additional file 2: Figure S2).

Furthermore, a Transwell assay was performed in these cells to evaluate the role of SPOP in mediating C/EBPα-regulated cell migration and invasion. The results indicated that the C/EBPα overexpression weakened the migration and invasion capacities of A427 cells and that this effect was reversed by the siSPOP transfection (Fig. 5c). Conversely, the attenuated migration and invasion abilities of the H1299 cells upon the C/EBPα depletion were restored by the SPOP expression plasmid transfection (Fig. 5d). In conclusion, our results indicate that SPOP is essential for the C/EBPα-regulated suppression of migration and invasion in lung cancer cells.

To further investigate the effects of SPOP on the C/EBPα-regulated progression of tumors, animal experiments were performed via subcutaneous transplantation of NSCLC cells into BALB/c nude mice. The results demonstrated that the C/EBPα depletion significantly promoted xenograft tumor growth. These effects were reversed by the SPOP overexpression (Fig. 5e, f). These data also show that SPOP is essential for the C/EBPα-regulated suppression of tumor growth in vivo.

## Discussion

Recently, SPOP has attracted increasing attention due to its role in tumorigenesis. Structurally, as an E3 ubiquitin ligase adaptor, SPOP contains an N-terminal MATH domain for substrate binding, a BTB domain for Cul3 binding, a C-terminal nuclear localization sequence and a 3-box domain [30]. Selectively, SPOP recruits substrates through its N-terminal MATH domain, while its BTB domain mediates its interaction and dimerization with Cul3 [31]. Recent studies have proven that SPOP is associated with the degradation and ubiquitination of several substrates, including CHOP, AR, steroid receptor coactivator SRC-3, ERα, death domain associate protein (Daxx), Gli, and so on, through which SPOP exerts its effects [32–34]. Some researchers have indicated that SPOP acts as a tumor suppressor in endometrial prostate and breast cancers. For example, SPOP acts as a cancer suppressor in prostate cancer by promoting the proteasomal degradation and ubiquitination of the SRC-3 (p160 steroid receptor coactivator-3) protein, thus suppressing androgen receptor transcription activity. This tumor suppressor effect is abrogated by prostate cancer associated SPOP mutations [35]. Conversely, Liu et al. demonstrated that SPOP is highly expressed in the majority of clear cell renal cell carcinomas, suggesting that SPOP plays a tumor promoting role in kidney cancer [36]. The conflictive effects of SPOP in different cells can be partially explained by the wide spectrum of substrates and SPOP differential expression in diverse cancer types. Furthermore, the limited studies suggest that SPOP performance in tumorigenesis is paradoxical, prompting us to search for more evidence. Although several studies investigating the relationship between SPOP and cancer have been performed, knowledge regarding the role of SPOP in lung cancer is limited.

The aberrant low SPOP expression may be attributed to epigenetic mechanisms. Some studies have identified the genetic alterations involved in the tumorigenesis of the SPOP gene, while few studies have explored epigenetic alterations. Numerous studies suggest that the hypermethylation of specific genes, mainly tumor suppressor genes, is associated with the onset and progression of lung cancer [37, 38]. Thus, our study focused on the SPOP methylation status in NSCLC and its correlation with the gene expression level. Our study found that the CpG sites in the SPOP promoter region were hypermethylated in NSCLC tissues and A549, A427 and H1299 lung cancer cells but hypomethylated in adjacent non-tumor tissues and 16HBE cells. Therefore, the expression of the SPOP gene in tumor cells involving a hypermethylated promoter is most likely repressed. Subsequently, we investigated the associations between SPOP gene methylation and clinicopathological variables in NSCLC patients. The methylation of the SPOP gene was only associated with the pathology grade of NSCLC, suggesting that the SPOP gene could play a more significant role in NSCLC tissue differentiation. However, gender, age, histologic type, smoking status, staging and lymph node metastasis were not significantly associated with SPOP gene methylation in NSCLC tissues. The pathology grade is an important factor for the prognosis of NSCLC. Therefore, SPOP gene methylation may be associated with poor NSCLC patient outcomes and can be a potential predictive biomarker for prognosis.

The process by which methylation regulates gene expression is reversible because the methylation status can be reversed by using a methyltransferase inhibitor [39]. We treated hypermethylated cell lines (A427 and H-1299 cells) with AZA, which is a well-known DNMT inhibitor, to confirm that CpG island methylation was indeed responsible for the decreased SPOP expression. AZA could elicit a dose-dependent induction of SPOP expression in the highly methylated A427 and H1299 cells.

In addition, after treatment with AZA at increasing concentrations, the mRNA and protein expression levels of the SPOP gene gradually increased, corresponding to the decreased methylation of the SPOP gene promoters. Subsequently, we found that epigenetically silenced SPOP mRNA and protein expression gradually reactivates after treatment with AZA at an increasing concentration. These results demonstrate that the hypermethylation of the SPOP gene promoter directly contributes to SPOP silencing in NSCLC cells. Furthermore, the demethylation agent AZA could relieve the SPOP promoter methylation, leading to increased SPOP expression and inhibiting cell growth in NSCLC.

DNA methylation participates in gene transcription via two major aspects. First, methylated DNA and methyl-CpG binding domain (MBD) proteins can recruit some chromatin remodeling proteins and histone deacetylases to the locus, leading to changes in the chromatin structure. Second, methylated cytosine residues can prevent transcription factors from binding their binding elements in the promoter region [40]. We demonstrated that C/EBPα is an important SPOP transcriptional regulator and that CpG site methylation within the C/EBPα binding element interfered with the binding affinity. The ChIP results demonstrated that C/EBPα clearly binds the SPOP gene promoter, and the sequential deletion and substitution mutation analyses demonstrated that three sites (− 1144 to − 1135, − 815 to − 806 and + 55 to + 64) are essential for C/EBPα-regulated SPOP gene promoter activity. Furthermore, the inhibition of the expression of the transcription factor C/EBPα can significantly reduce SPOP expression and ultimately affect cell migration, invasion and proliferation and tumor growth. These data indicate that DNA methylation can affect the binding of the transcription factor C/EBPα to the SPOP gene promoter and then participate in the regulation of SPOP gene expression.

## Conclusions

Our study is the first to show that the CpG island in the SPOP gene promoter in NSCLC is hypermethylated and that abnormal methylation is only associated with the pathology state of NSCLC. Furthermore, the DNA methyltransferase inhibitor AZA could decrease SPOP promoter region hypermethylation and recover gene activity, leading to the increased expression of SPOP. Therefore, the detection of SPOP methylation could be beneficial for the early diagnosis of NSCLC, and the SPOP methylation status may be a valuable prognostic biomarker in NSCLC. This study also found that the down-regulation of SPOP expression is mediated by suppressed C/EBPα binding to the hypermethylated promoter of SPOP, which may be involved in NSCLC occurrence. Our study found that the SPOP promoter methylation status can be utilized as an epigenetic biomarker of NSCLC and that the C/EBPα-SPOP signaling pathway can be a potential target for NSCLC prevention and treatment.

## Additional files

**Additional file 1: Figure S1.** Correlation between C/EBPα and SPOP gene expression based on the TCGA database. (A, B) The expression of C/EBPα and SPOP in lung adenocarcinoma and lung squamous cell carcinoma has a downward trend compared with that in normal tissues. (C, D) Correlation analysis showing that the alterations in C/EBPα and SPOP were significantly positively correlated.

**Additional file 2: Figure S2.** SPOP mediates C/EBPα-regulated proliferation of lung cancer cells. (A, B) The cell proliferation abilities were assessed by MTT assays in H1299 (A) and A429 (B) cells treated as indicated. The data are presented as the mean ± SD of three independent experiments. *P < 0.05 versus H1299 or A427 cells cotransfected with siRNA and vector control (siControl + Vector); ^#^P < 0.05 versus H1299 cells cotransfected with siSPOP and vector control (Vector + siSPOP) or A427 cells cotransfected with siControl and SPOP expression plasmid (siControl + SPOP).

## Abbreviations

NSCLC: non-small cell lung cancer
SPOP: speckle-type POZ protein
TSG: cancer suppressor gene
C/EBPα: C/EBP alpha
FBS: fetal bovine serum
MSP: methylation-specific PCR
BSP: bisulfite sequencing PCR
qRT-PCR: quantitative real-time PCR
OD: optical density
AZA: 5-aza-2′-deoxycytidine
ChIP: chromatin immunoprecipitation
MBD: methyl-CpG binding domain

## References

[1] Siegel R, Ma J, Zou Z, Jemal A (2014). Cancer statistics, 2014. CA Cancer J Clin.

[2] Ali G, Donati V, Loggini B, Servadio A, Dell’Omodarme M, Prati MC, Camacci T, Lucchi M, Melfi F, Mussi A (2008). Different estrogen receptor beta expression in distinct histologic subtypes of lung adenocarcinoma. Hum Pathol.

[3] Pastorino U (2010). Lung cancer screening. Br J Cancer.

[4] Liu SV, Giaccone G (2014). Lung cancer in 2013: refining standard practice and admitting uncertainty. Nat Rev Clin Oncol.

[5] Kan Z, Jaiswal BS, Stinson J, Janakiraman V, Bhatt D, Stern HM, Yue P, Haverty PM, Bourgon R, Zheng J (2010). Diverse somatic mutation patterns and pathway alterations in human cancers. Nature.

[6] McMahon KW, Karunasena E, Ahuja N (2017). The roles of DNA methylation in the stages of cancer. Cancer J.

[7] Knutson SK, Wigle TJ, Warholic NM, Sneeringer CJ, Allain CJ, Klaus CR, Sacks JD, Raimondi A, Majer CR, Song J (2012). A selective inhibitor of EZH2 blocks H3K27 methylation and kills mutant lymphoma cells. Nat Chem Biol.

[8] Song X, Zhang L, Gao T, Ye T, Zhu Y, Lei Q, Feng Q, He B, Deng H, Yu L (2016). Selective inhibition of EZH2 by ZLD10A blocks H3K27 methylation and kills mutant lymphoma cells proliferation. Biomed Pharmacother.

[9] Helin K, Dhanak D (2013). Chromatin proteins and modifications as drug targets. Nature.

[10] Fasanelli F, Baglietto L, Ponzi E, Guida F, Campanella G, Johansson M, Grankvist K, Johansson M, Assumma MB, Naccarati A (2015). Hypomethylation of smoking-related genes is associated with future lung cancer in four prospective cohorts. Nat Commun.

[11] Nagai Y, Kojima T, Muro Y, Hachiya T, Nishizawa Y, Wakabayashi T, Hagiwara M (1997). Identification of a novel nuclear speckle-type protein, SPOP. FEBS Lett.

[12] Aravind L, Koonin EV (1999). Fold prediction and evolutionary analysis of the POZ domain: structural and evolutionary relationship with the potassium channel tetramerization domain. J Mol Biol.

[13] Nakayama KI, Nakayama K (2006). Ubiquitin ligases: cell-cycle control and cancer. Nat Rev Cancer.

[14] Singer JD, Gurian-West M, Clurman B, Roberts JM (1999). Cullin-3 targets cyclin E for ubiquitination and controls S phase in mammalian cells. Genes Dev.

[15] Genschik P, Sumara I, Lechner E (2013). The emerging family of CULLIN3-RING ubiquitin ligases (CRL3 s): cellular functions and disease implications. EMBO J.

[16] Zhuang M, Calabrese MF, Liu J, Waddell MB, Nourse A, Hammel M, Miller DJ, Walden H, Duda DM, Seyedin SN (2009). Structures of SPOP-substrate complexes: insights into molecular architectures of BTB-Cul3 ubiquitin ligases. Mol Cell.

[17] Chen HY, Chen RH (2016). Cullin 3 ubiquitin ligases in cancer biology: functions and therapeutic implications. Front Oncol.

[18] Barbieri CE, Baca SC, Lawrence MS, Demichelis F, Blattner M, Theurillat JP, White TA, Stojanov P, Van Allen E, Stransky N (2012). Exome sequencing identifies recurrent SPOP, FOXA1 and MED12 mutations in prostate cancer. Nat Genet.

[19] Le Gallo M, O’Hara AJ, Rudd ML, Urick ME, Hansen NF, O’Neil NJ, Price JC, Zhang S, England BM, Godwin AK (2012). Exome sequencing of serous endometrial tumors identifies recurrent somatic mutations in chromatin-remodeling and ubiquitin ligase complex genes. Nat Genet.

[20] Kim MS, Je EM, Oh JE, Yoo NJ, Lee SH (2013). Mutational and expressional analyses of SPOP, a candidate tumor suppressor gene, in prostate, gastric and colorectal cancers. (Acta Pathologica Microbiologica et Immunologica Scandinavica) APMIS.

[21] Kim MS, Kim MS, Yoo NJ, Lee SH (2014). Somatic mutation of SPOP tumor suppressor gene is rare in breast, lung, liver cancers, and acute leukemias. (Acta Pathologica Microbiologica et Immunologica Scandinavica) APMIS.

[22] Li JJ, Zhang JF, Yao SM, Huang H, Zhang S, Zhao M, Huang JA (2017). Decreased expression of speckle-type POZ protein for the prediction of poor prognosis in patients with non-small cell lung cancer. Oncol Lett.

[23] Oakes CC, Claus R, Gu L, Assenov Y, Hullein J, Zucknick M, Bieg M, Brocks D, Bogatyrova O, Schmidt CR (2014). Evolution of DNA methylation is linked to genetic aberrations in chronic lymphocytic leukemia. Cancer Discov.

[24] Hohaus S, Petrovick MS, Voso MT, Sun Z, Zhang DE, Tenen DG (1995). PU1 (Spi-1) and C/EBP alpha regulate expression of the granulocyte-macrophage colony-stimulating factor receptor alpha gene. Mol Cell Biol..

[25] Lefterova MI, Zhang Y, Steger DJ, Schupp M, Schug J, Cristancho A, Feng D, Zhuo D, Stoeckert CJ, Liu XS (2008). PPARgamma and C/EBP factors orchestrate adipocyte biology via adjacent binding on a genome-wide scale. Genes Dev.

[26] Pan Z, Zheng W, Zhang J, Gao R, Li D, Guo X, Han H, Li F, Qu S, Shao R (2014). Down-regulation of the expression of CCAAT/enhancer binding protein alpha gene in cervical squamous cell carcinoma. BMC Cancer.

[27] Huan H, Wen X, Chen X, Wu L, Liu W, Habib NA, Bie P, Xia F (2016). C/EBPalpha short-activating RNA suppresses metastasis of hepatocellular carcinoma through inhibiting EGFR/beta-catenin signaling mediated EMT. PLoS ONE.

[28] Burke E, Barik S (2003). Megaprimer PCR: application in mutagenesis and gene fusion. Methods Mol Biol.

[29] Lourenco AR, Coffer PJ (2017). A tumor suppressor role for C/EBPα in solid tumors: more than fat and blood. Oncogene.

[30] Mani RS (2014). The emerging role of speckle-type POZ protein (SPOP) in cancer development. Drug Discov Today.

[31] Gao K, Jin X, Tang Y, Ma J, Peng J, Yu L, Zhang P, Wang C (2015). Tumor suppressor SPOP mediates the proteasomal degradation of progesterone receptors (PRs) in breast cancer cells. Am J Cancer Res.

[32] Kwon JE, La M, Oh KH, Oh YM, Kim GR, Seol JH, Baek SH, Chiba T, Tanaka K, Bang OS (2006). BTB domain-containing speckle-type POZ protein (SPOP) serves as an adaptor of Daxx for ubiquitination by Cul3-based ubiquitin ligase. J Biol Chem.

[33] Li C, Ao J, Fu J, Lee DF, Xu J, Lonard D, O’Malley BW (2011). Tumor-suppressor role for the SPOP ubiquitin ligase in signal-dependent proteolysis of the oncogenic co-activator SRC-3/AIB1. Oncogene.

[34] Zhang P, Gao K, Jin X, Ma J, Peng J, Wumaier R, Tang Y, Zhang Y, An J, Yan Q (2015). Endometrial cancer-associated mutants of SPOP are defective in regulating estrogen receptor-alpha protein turnover. Cell Death Dis.

[35] Geng C, He B, Xu L, Barbieri CE, Eedunuri VK, Chew SA, Zimmermann M, Bond R, Shou J, Li C (2013). Prostate cancer-associated mutations in speckle-type POZ protein (SPOP) regulate steroid receptor coactivator 3 protein turnover. Proc Natl Acad Sci USA.

[36] Liu J, Ghanim M, Xue L, Brown CD, Iossifov I, Angeletti C, Hua S, Negre N, Ludwig M, Stricker T (2009). Analysis of Drosophila segmentation network identifies a JNK pathway factor overexpressed in kidney cancer. Science.

[37] Brzezianska E, Dutkowska A, Antczak A (2013). The significance of epigenetic alterations in lung carcinogenesis. Mol Biol Rep.

[38] Van Den Broeck A, Ozenne P, Eymin B, Gazzeri S (2010). Lung cancer: a modified epigenome. Cell Adhes Migr.

[39] Park JY, Kim D, Yang M, Park HY, Lee SH, Rincon M, Kreahling J, Plass C, Smiraglia DJ, Tockman MS (2013). Gene silencing of SLC5A8 identified by genome-wide methylation profiling in lung cancer. Lung Cancer.

[40] Tate PH, Bird AP (1993). Effects of DNA methylation on DNA-binding proteins and gene expression. Curr Opin Genet Dev.

