# Supplementary figures and images for: Speckle-type POZ protein functions as a tumor suppressor in non-small cell lung cancer due to DNA methylation
Source: Cancer Cell Int. 2018 Dec 22;18:213. doi: 10.1186/s12935-018-0711-z (PMC6304003; doi:10.1186/s12935-018-0711-z)

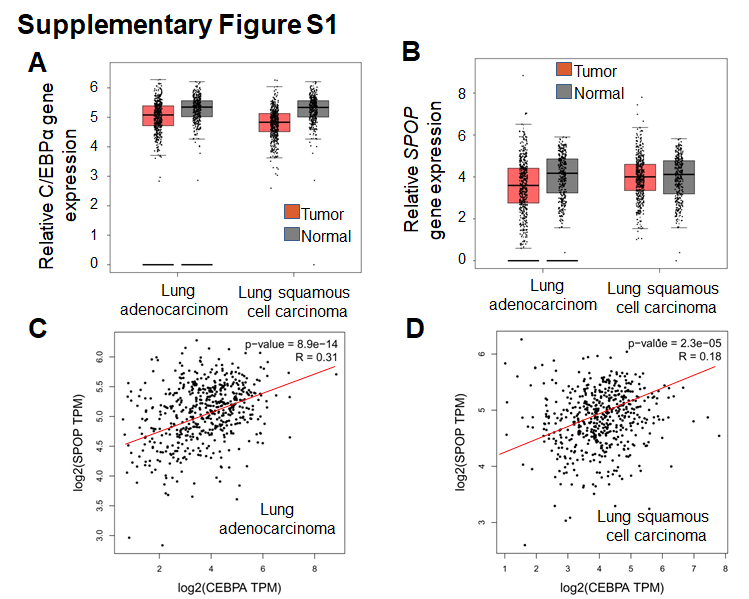

Supplement: Supplementary file 1 — Additional file 1: Figure S1. Correlation between C/EBPα and SPOP gene expression based on the TCGA database. (A, B) The expression of C/EBPα and SPOP in lung adenocarcinoma and lung squamous cell carcinoma has a downward trend compared with that in normal tissues. (C, D) Correlation analysis showing that the alterations in C/EBPα and SPOP were significantly positively correlated. [file 12935_2018_711_MOESM1_ESM.tif]

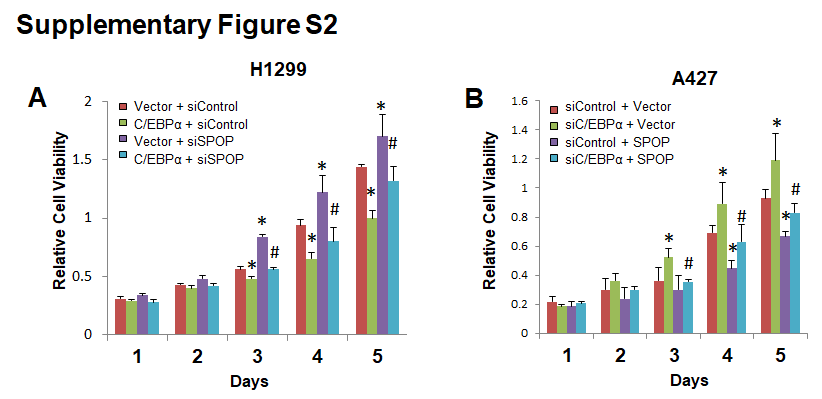

Supplement: Supplementary file 2 — Additional file 2: Figure S2. SPOP mediates C/EBPα-regulated proliferation of lung cancer cells. (A, B) The cell proliferation abilities were assessed by MTT assays in H1299 (A) and A429 (B) cells treated as indicated. The data are presented as the mean ± SD of three independent experiments. *P < 0.05 versus H1299 or A427 cells cotransfected with siRNA and vector control (siControl + Vector); #P < 0.05 versus H1299 cells cotransfected with siSPOP and vector control (Vector + siSPOP) or A427 cells cotransfected with siControl and SPOP expression plasmid (siControl + SPOP). [file 12935_2018_711_MOESM2_ESM.tif]
